# Amygdalin Exerts Antitumor Activity in Taxane-Resistant Prostate Cancer Cells

**DOI:** 10.3390/cancers14133111

**Published:** 2022-06-24

**Authors:** Igor Tsaur, Anita Thomas, Michelle Monecke, Marion Zugelder, Jochen Rutz, Timothy Grein, Sebastian Maxeiner, Hui Xie, Felix K.-H. Chun, Florian Rothweiler, Jindrich Cinatl, Martin Michaelis, Axel Haferkamp, Roman A. Blaheta

**Affiliations:** 1 Department of Urology and Pediatric Urology, University Medicine Mainz, Langenbeckstr. 1, 55131 Mainz, Germany; igor.tsaur@unimedizin-mainz.de (I.T.); axel.haferkamp@unimedizin-mainz.de (A.H.); roman.blaheta@unimedizin-mainz.de (R.A.B.); 2 Department of Urology, Goethe-University, 60590 Frankfurt am Main, Germany; michellemonecke@gmail.com (M.M.); marion@zugelder.org (M.Z.); jochen.Rutz@kgu.de (J.R.); Timothy.Grein@kgu.de (T.G.); sebastian.maxeiner@kgu.de (S.M.); xiehui0831@outlook.com (H.X.); felix.chun@kgu.de (F.K.-H.C.); 3 Institute of Medical Virology, Goethe-University, 60596 Frankfurt am Main, Germany; f.rothweiler@kinderkrebsstiftung-frankfurt.de (F.R.);; 4 Petra Joh-Forschungshaus, 60528 Frankfurt am Main, Germany; 5 Industrial Biotechnology Centre, School of Biosciences, University of Kent, Canterbury CT2 7NJ, UK; m.michaelis@kent.ac.uk

**Keywords:** prostate cancer, resistant cell lines, complementary/alternative medicine (CAM), amygdalin, docetaxel, cabozantinib, cabazitaxel

## Abstract

**Simple Summary:**

The natural compound amygdalin is popular among tumor patients as an alternative treatment option. However, knowledge about its precise mode of action remains poor. In the present study, amygdalin is shown to inhibit growth and disseminative properties of taxane-resistant prostate cancer cells. Further investigation is warranted to determine the role of amygdalin in the setting of metastasized prostate cancer.

**Abstract:**

Despite recent advances in the treatment of metastatic prostate cancer (PCa), resistance development after taxane treatments is inevitable, necessitating effective options to combat drug resistance. Previous studies indicated antitumoral properties of the natural compound amygdalin. However, whether amygdalin acts on drug-resistant tumor cells remains questionable. An in vitro study was performed to investigate the influence of amygdalin (10 mg/mL) on the growth of a panel of therapy-naïve and docetaxel- or cabazitaxel-resistant PCa cell lines (PC3, DU145, and LNCaP cells). Tumor growth, proliferation, clonal growth, and cell cycle progression were investigated. The cell cycle regulating proteins (phospho)cdk1, (phospho)cdk2, cyclin A, cyclin B, p21, and p27 and the mammalian target of rapamycin (mTOR) pathway proteins (phospho)Akt, (phospho)Raptor, and (phospho)Rictor as well as integrin β1 and the cytoskeletal proteins vimentin, ezrin, talin, and cytokeratin 8/18 were assessed. Furthermore, chemotactic activity and adhesion to extracellular matrix components were analyzed. Amygdalin dose-dependently inhibited tumor growth and reduced tumor clones in all (parental and resistant) PCa cell lines, accompanied by a G0/G1 phase accumulation. Cell cycle regulating proteins were significantly altered by amygdalin. A moderate influence of amygdalin on tumor cell adhesion and chemotaxis was observed as well, paralleled by modifications of cytoskeletal proteins and the integrin β1 expression level. Amygdalin may, therefore, block tumor growth and disseminative characteristics of taxane-resistant PCa cells. Further studies are warranted to determine amygdalin’s value as an antitumor drug.

## 1. Introduction

Accounting for 1.3 million incident cases and 416,000 deaths, prostate cancer (PCa) was the 3rd most common neoplasm and the 5th leading cause of cancer deaths in 2017 worldwide, contributing to 7.1 million of disability-adjusted life years, with 88% coming from years of life lost and 12% from years lived with disability [1]. The incidence of PCa increased by a substantial 42% between 2007 and 2017, whereas the odds of developing this malignancy is currently 1 in 18 [1]. Alarmingly, the absolute number of annual new cases of metastatic PCa (mPCa) in the US was forecasted to rise by 42% over the next decade in the period 2015–2025 [2]. The resultant socioeconomic burden is formidable, since treatment costs of PCa are increasing more rapidly than those of any other tumor [3].

Growing healthcare expenditures are considerably attributable to several anticancer drugs approved for mPCa therapy in the last decade due to their efficacy for survival prolongation and improvement of quality of life [4]. While abiraterone acetate, enzalutamide, docetaxel, and cabazitaxel along with androgen deprivation therapy (ADT) were all the mainstay of systemic management of castration-resistant mPCa (mCRPC) for years, the upfront use of one of the first three compounds or apalutamide with concurrent ADT in hormone-sensitive mPCa (mHSPC) has revolutionized the treatment paradigm of this stage of disease [5]. While clinical effectiveness of either of the aforementioned approaches related to the extension of overall survival in mHSPC was similar in several indirect comparisons, further aspects, such as tumor characteristics, patient preferences, cost-effectiveness, and toxicity, should be taken into account for personalized decision making [5,6]. To this end, docetaxel and cabazitaxel seem to be cost-effective; however, they are more toxic for mPCa management when compared to antihormonal agents [4,5,7].

Despite an impressive activity in mPCa, disease progression is unavoidable after taxane treatment, necessitating the development of novel approaches conquering emerging drug resistance. Particularly in light of common taxane-related side effects during previous therapy [8], tolerability of the next-line approach is critical. In this context, amygdalin, a cyanogenic glycoside existing in seeds and kernels of some fruits and exerting antiproliferative, antioxidative, and immunoregulatory activities, might represent an intriguing option for mPCa treatment [9,10]. Importantly, no relevant toxicity for purified amygdalin utilized in “therapeutic” dosing has been reported yet [11]. Thus, the aim of our investigation was to assess anticancer activity of amygdalin in docetaxel- and cabazitaxel-resistant PCa cells.

## 2. Materials and Methods

### 2.1. Cell Cultures

Human, castration-resistant prostate tumor cell lines PC3 and DU145 and castration-sensitive LNCaP cells were obtained from the German Collection of Microorganisms and Cell Cultures (DSMZ). The resistant sublines were derived from the Resistant Cancer Cell Line (RCCL) collection [12]. Cells were grown and subcultured in RPMI 1640 medium (Gibco/Invitrogen, Karlsruhe, Germany) and supplemented with 10% fetal calf serum (FCS) (Gibco/Invitrogen, Karlsruhe, Germany), 2% HEPES buffer (Sigma-Aldrich, Darmstadt, Germany), 2% glutamine (Gibco/Invitrogen, Karlsruhe, Germany), and 1% penicillin/streptomycin (Gibco/Invitrogen, Karlsruhe, Germany) at 37 °C in a humidified, 5% CO_2_ incubator.

### 2.2. Induction of Drug Resistance and Drug Treatment

Resistant sublines were established by continuous exposure to increasing concentrations of the respective drugs, as described before [13]. The docetaxel-resistant tumor cells were exposed to 2.5 ng/mL docetaxel (Sanofi, Paris, France), and cabazitaxel-resistant tumor cells were exposed to 2.5 ng/mL cabazitaxel (Sanofi, Paris, France) three times a week.

Amygdalin from apricot kernels (Sigma-Aldrich, Taufkirchen, Germany) was freshly dissolved in cell culture medium and added to tumor cells at 10 mg/mL based on earlier studies [14]. Controls remained untreated. In all experiments, treated tumor cell cultures were compared to nontreated cultures. To assess toxic effects of amygdalin and/or docetaxel and cabazitaxel, cell viability was determined by trypan blue (Gibco/Invitrogen).

### 2.3. Cell Growth

Cell growth was measured using a 3-(4,5-dimethylthiazol-2-yl)-2,5-diphenyltetrazolium bromide (MTT) dye (Roche Diagnostics, Penzberg, Germany). PCa cells (100 µL, 1 × 10^4^ cells/mL) were seeded onto 96-well tissue culture plates containing serial dilutions of amygdalin. Controls were incubated without amygdalin. After 24, 48, and 72 h, MTT (0.5 mg/mL) was added for 4 h. Subsequently, cells were lysed in a buffer containing 10% SDS in 0.01 M HCl. The plates then were incubated overnight at 37 °C, 5% CO_2_. Absorbance at 550 nm was detected for each well using a microplate enzyme-linked immunosorbent assay (ELISA) reader. Cell number after 24 h incubation was set to 100% in order to illustrate the kinetics of dose response.

### 2.4. Cell Proliferation

Cell proliferation was measured using a BrdU (5-bromo-2′-deoxyuridine) cell proliferation ELISA kit (Calbiochem/Merck Biosciences, Darmstadt, Germany).

Tumor cells seeded onto 96-well plates (5000/well), were incubated with 20 µL BrdU-labeling solution per well for 24 h, and were then fixed and stained using antiBrdU mAb. Unspecific background values, evaluated by incubating the tumor cells without BrdU, were subtracted. Absorbance was detected at 450 nm using a microplate ELISA reader. Values were presented as % compared to untreated controls set to 100%.

### 2.5. Clonogenic Assay

A total of 500 PCa cells treated with 10 mg/mL amygdalin per well were transferred to 6-well plates. Untreated PCa cells served as controls. After 10 days incubation without medium change, cell colonies were fixed and counted. A colony was defined as consisting of at least 50 cells. Untreated controls were set to 100%.

### 2.6. Cell Cycle Phase Distribution

Cell cycle analysis was carried out on subconfluent cells. Tumor cell populations were stained with propidium iodide, using a CycleTEST PLUS DNA Reagent Kit and then subjected to flow cytometry using FACScan (both from Becton-Dickinson, Heidelberg, Germany). A total of 10,000 events were collected from each sample. Data acquisition was carried out using CellQuest software, and cell cycle distribution was analyzed by ModFit software (Becton-Dickinson). The number of cells in G0/1, G2/M, or S phases was expressed as the percentage of the total cell number.

### 2.7. Adhesion to Extracellular Matrix Components

Six-well plates were coated with collagen G (obtained from calf skin, 90% collagen type I, and 10% collagen type III, Biochrom, Berlin, Germany; diluted to 400 µg/mL in phosphate-buffered saline (PBS)) or fibronectin (obtained from human plasma, BD Biosciences, diluted to 50 µg/mL in PBS) and laminin (obtained from the Engelbreth–Holm–Swarm mouse tumor, BD Biosciences diluted to 50 µg/mL in PBS) overnight at 4 °C. Plastic dishes were used as the background control. To block nonspecific cell adhesion, plates were washed with 1% bovine serum albumin (BSA) in PBS. Subsequently, 0.5 × 10^6^ PCa cells, treated with culture medium alone or pretreated with amygdalin, were added to each well for 1 h. Then, nonadherent cells were fixed with 1% glutaraldehyde and counted. The mean cellular adhesion rate (number of cells adherent to the coated wells minus number of cells adherent to the noncoated wells) was determined from five different observation fields (5 × 0.25 mm^2^).

### 2.8. Chemotactic Migration

Serum-induced chemotactic movement was assessed by six-well (8-µm pores) Transwell chambers (Greiner, Frickenhausen. Germany) with PCa cells that were pretreated with amygdalin for 72 h. A total of 0.5 × 10^6^ PC3 or DU145 cells per mL (LNCaP did not migrate and, thus, were excluded from the chemotaxis assay) were then placed in the upper chamber in serum-free medium, either free of amygdalin or containing amygdalin. The lower chamber contained 10% serum providing the serum gradient necessary for tumor cell migration. After 20 h incubation, the nonmigrating cells on upper surface of the Transwell membrane were gently removed with a cotton swab. Cells that had migrated to the lower surface of the membrane were stained and counted. The mean chemotaxis rate was determined from five different observation fields.

### 2.9. Western Blot Analysis

PCa cell lysates were applied to a polyacrylamide gel and electrophoresed for 1 h at 100 V. The protein was then transferred to a nitrocellulose membrane (1 h, 100 V), which was then blocked by nonfat dry milk for 1 h. The membranes were then incubated overnight with following primary antibodies directed against the cell cycle regulating proteins: p21 (IgG1, clone 2G12), p27 (IgG1, clone G173-524), CDK1/Cdc2 (IgG1, clone 1), pCDK1/Cdc2 (IgG1, clone 44/CDK1/Cdc2 (pY15)), CDK2 (IgG2a, clone 55), Cyclin A (IgG1, clone 25), Cyclin B (IgG1, clone 18) (all obtained from BD Biosciences), and pCDK2 (Thr160 Cell Signaling).

The mechanistic target of rapamycin (mTOR) pathway was investigated using the following monoclonal antibodies: Raptor (24C12 Cell Signaling), pRaptor (IgG, Ser792), Rictor (D16H9; Cell Signaling), pRictor (IgG, Thr1135, clone D30A3; all from Cell Signaling), PKBα/Akt (IgG1 clone 55), and anti-phospho-Akt (pAkt; IgG1, Ser472/Ser473, clone 104A282; both: BD Pharmingen). To indicate cytoskeletal proteins and integrin β1, the following primary antibodies were used: Vimentin (IgG; clone D21H3; Cell Signaling, Frankfurt, Germany), ezrin (polyclonal), talin 1 (rabbit IgG, clone C45F1), cytokeratin 8/18 (mouse IgG1, clone C51, all: Cell Signaling, Leiden, the Netherlands), and integrin β1 (mouse IgG1, 1:2500, clone 18; #610468, BD Biosciences, Heidelberg, Germany).

HRP-conjugated goat antimouse and antirabbit IgG (both 1:5000; Upstate Biotechnology, Lake Placid, NY, USA) served as secondary antibodies. Antibody complexes were detected using the enhanced chemiluminescence (ECL) detection reagent (ECLTM, Amersham/GE Healthcare, München, Germany) and then visualized by the Fusion FX7 system (Peqlab, Erlangen, Germany). Protein bands were normalized to β-actin (Sigma, Taufenkirchen, Germany; dilution 1:1000), which served as the internal control. Pixel density analysis of the protein bands (both total and phosphorylated) was achieved by calculating the ratio of protein intensity/β-actin intensity (GIMP 2.8 software, www.gimp.org, accessed on 15 June 2022).

### 2.10. Statistics

All experiments were performed at least 3 times, and statistical significance was determined with the Student’s t-test or Wilcoxon–Mann–Whitney U test. Differences were considered statistically significant at *p* < 0.05.

## 3. Results

### 3.1. Resistance Induction

**Figure 1:**
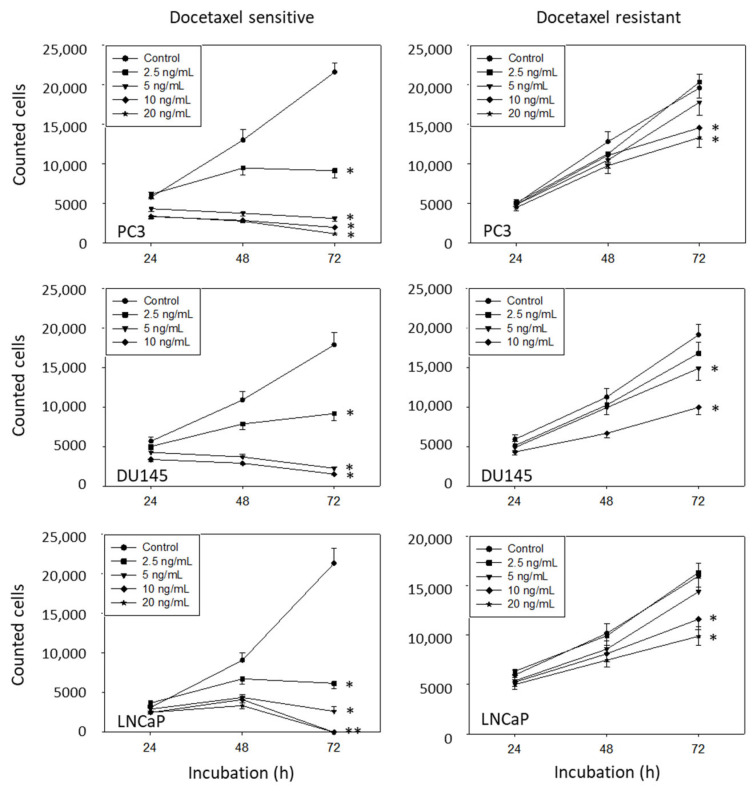

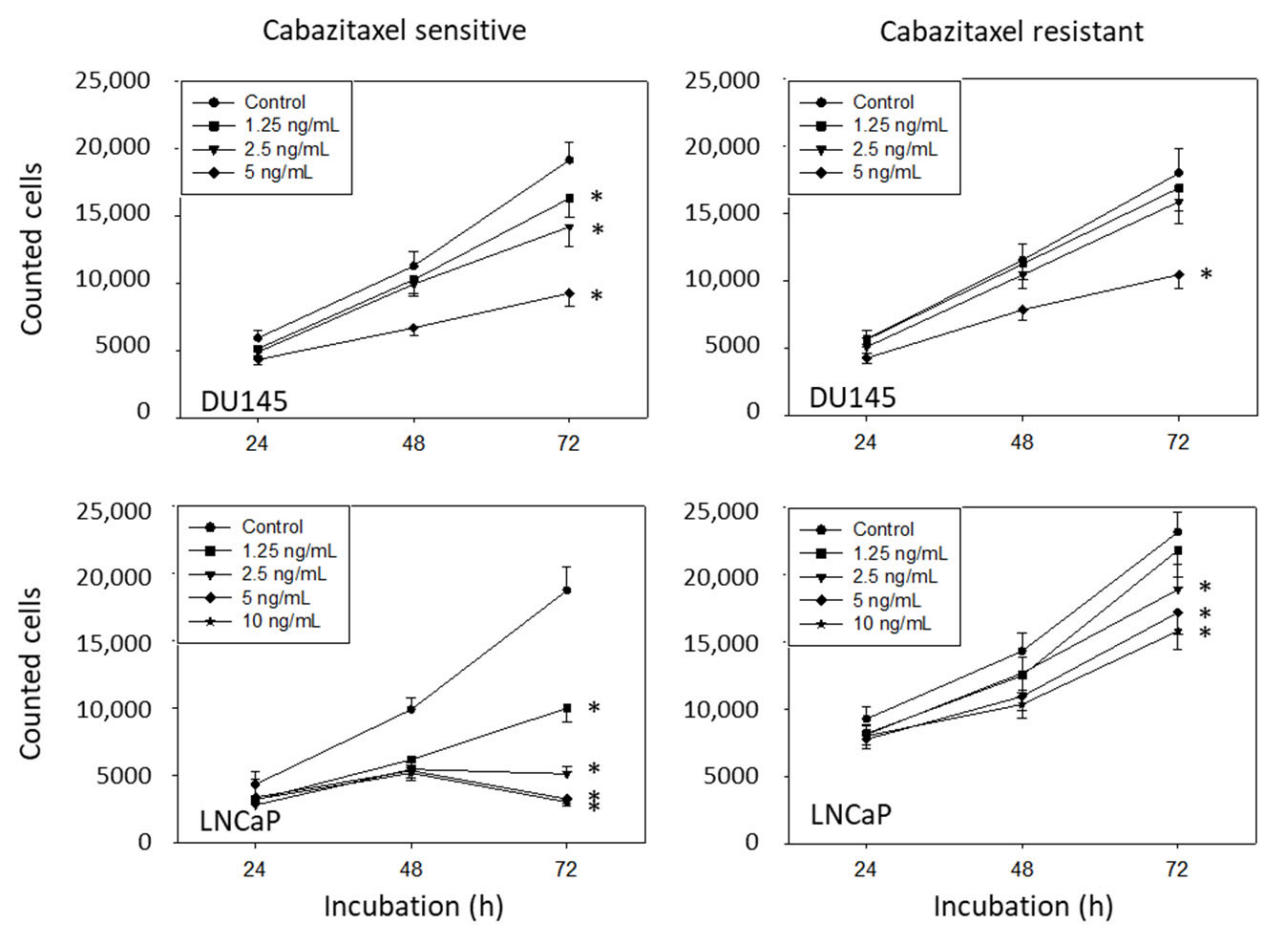
Dose–response analysis of sensitive and drug-resistant prostate cancer cells. Growth of cabazitaxel-sensitive versus cabazitaxel-resistant cells has not been evaluated on PC3 cells. Error bars indicate standard deviation (SD). * indicates significant difference to the corresponding control, n = 5.

The PCa cell lines were treated with gradually increasing concentrations of docetaxel (PC3, DU145, and LNCaP) or cabazitaxel (DU145 and LNCaP) to induce resistance. Figure 1 depicts the dose–response relationship of parental (sensitive) versus-resistant cells. Docetaxel, applied at 2.5 ng/mL, already caused a significant growth reduction of PC3, DU145, and LNCaP cells, whereas higher concentrations were necessary to (moderately) suppress growth of the drug-resistant cells. Cabazitaxel did not induce resistance in PC3 cells. However, low-dosed cabazitaxel (1.25 ng/mL) caused a significant growth blockade of DU145 and LNCaP cells, which was not the case in the respective resistant sublines. All further studies were carried out in the presence of 2.5 ng/mL docetaxel or 2.5 ng/mL cabazitaxel.

### 3.2. Amygdalin Blocks Growth and Proliferation of Resistant and Sensitive Tumor Cells

**Figure 2:**
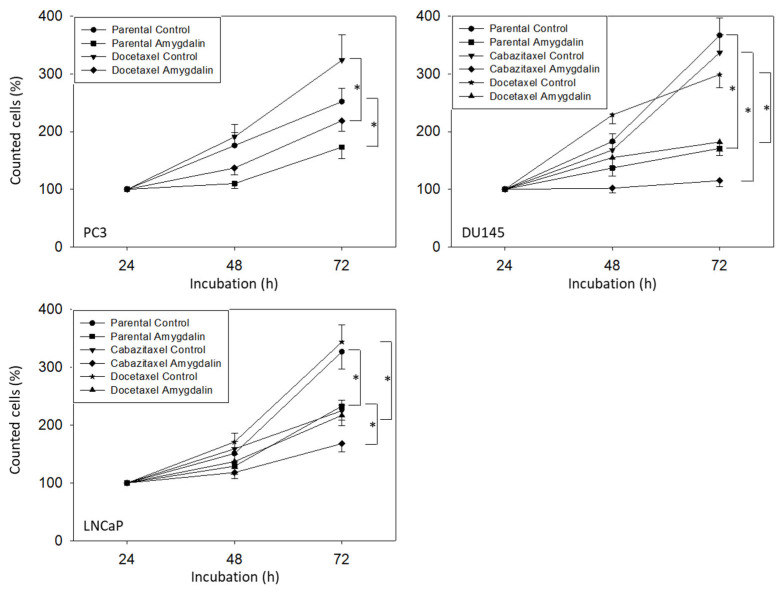
Influence of amygdalin on the growth of parental, cabazitaxel- and docetaxel-resistant PC3, DU145, and LNCaP cell lines. Cell count is related to the 24 h value set to 100%. Error bars indicate standard deviation (SD), n = 5. * indicates significant difference to the corresponding non-treated control.

Amygdalin, applied at 10 mg/mL, caused a significant downregulation of PC3, DU145, and LNCaP cell numbers, independent of whether they were resistant to cabazitaxel or docetaxel or not (Figure 2).

**Figure 3:**
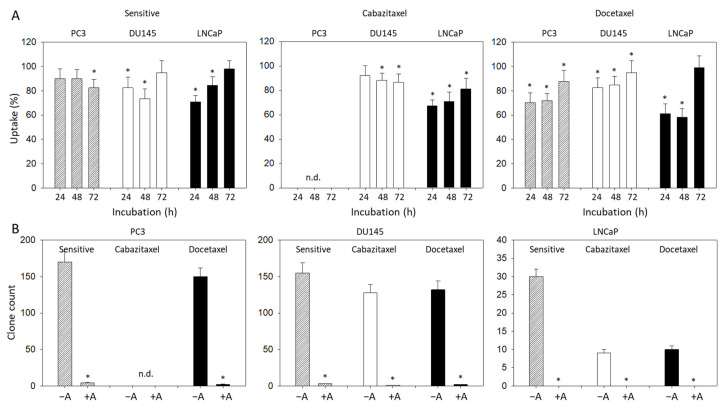
(**A**): BrdU uptake in sensitive and drug-resistant PC3, DU145, and LNCaP cells following amygdalin exposure for 24, 48, and 72 h. Values are given in percentage and are related to untreated controls, which were set to 100%. (**B**): Number of PC3, DU145, and LNCaP clones (drug-sensitive, cisplatin-resistant, and gemcitabine-resistant) exposed to amygdalin (+A), compared to the nontreated controls (−A). Error bars indicate standard deviation (SD), n = 3. * indicates significant difference to untreated controls. n.d.: not done.

Proliferation data (BrdU uptake) were inhomogeneous. Concerning the sensitive cell lines, a significant reduction of BrdU incorporation in the presence of amygdalin was noted after 72 h in PC3 but after 24 and 48 h in the DU145 and LNCaP cells (Figure 3A). Based on the docetaxel-resistant cells, the amygdalin-triggered effects were at a maximum after 24 and 48 h in all cell lines evaluated. Most importantly, a very prominent loss of PC3 and DU145 tumor clones was seen following amygdalin exposure (Figure 3B). No clones were recorded at all when LNCaP cells (both sensitive and resistant) were treated with amygdalin.

### 3.3. Cell Cycling and Cell Cycle Regulating Proteins

**Figure 4:**
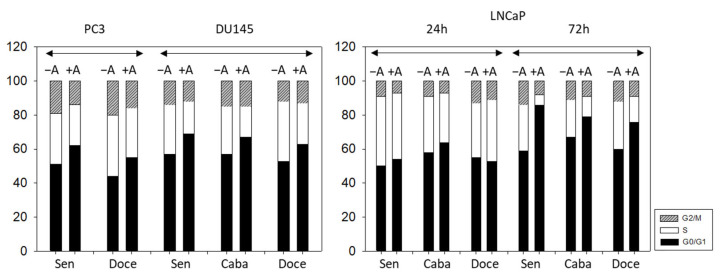
Cell cycle distribution in drug-sensitive and drug-resistant PC3, DU145, and LNCaP cells following amygdalin exposure (+A) for 24 (all cell lines) and 72 h (LNCaP cells). Controls (−A) remained untreated. One representative of three separate experiments is shown (n = 3).

Treatment of PC3 cells (sensitive and docetaxel-resistant) with amygdalin elevated the number of G0/G1 and reduced the number of G2/M phase cells (Figure 4). Elevation of G0/G1 phase cells was also observed when DU145 cells (sensitive, docetaxel-resistant, and cabazitaxel-resistant) were exposed to amygdalin, whereas the number of S phase cells decreased, each compared to the untreated controls. No distinct alterations of LNCaP were evoked after a 24 h amygdalin incubation. However, strong effects were seen after 72 h with a prominent upregulation of G0/G1 phase cells and a diminution of S phase cells. The effect was exerted on both sensitive and resistant sublines.

**Figure 5:**
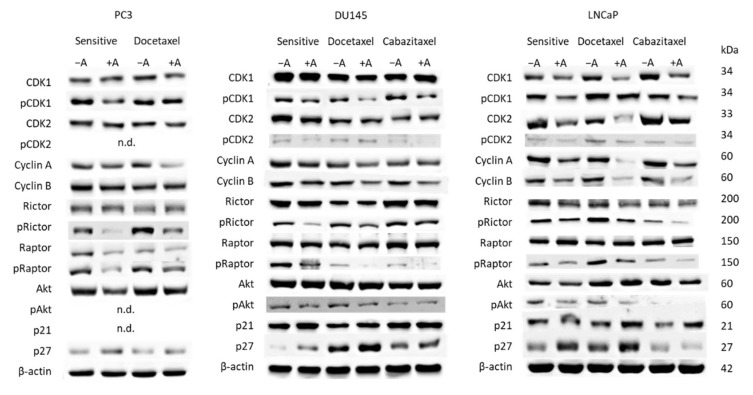

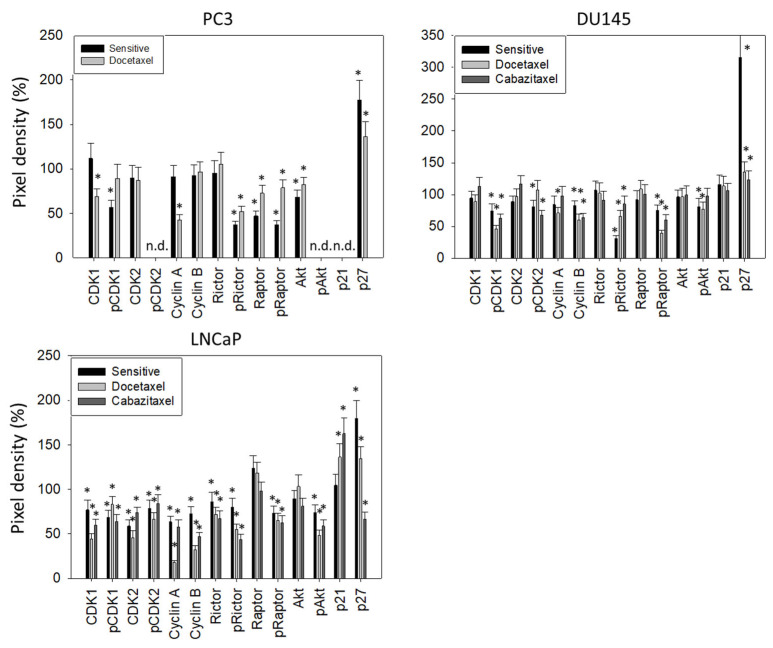
Up: Western blot of cell cycle and mTOR-related proteins from drug-sensitive and drug-resistant PC3, DU145, and LNCaP cells. Tumor cells received either amygdalin (+A) or cell culture medium alone (−A) for 24 h. β-actin served as the internal control. One representative from three separate experiments. n.d.: not done. Down: The ratio of protein intensity/β-actin intensity expressed as a percentage of the controls, set to 100%. Error bars indicate standard deviation, n = 3. * indicates significant difference to controls. n.d.: not done. Original blots see supplementary file S1.

Cell cycle regulating proteins were analyzed thereafter (Figure 5). Concerning PC3 cells, the following proteins became downregulated by amygdalin: CDK1 (docetaxel-resistant cells), pCDK1 (sensitive cells), CDK2 (docetaxel-resistant cells), Cyclin A (docetaxel-resistant cells), pRictor, Raptor, pRaptor, and Akt (sensitive cells > docetaxel-resistant cells). In contrast, p27 was upregulated by amygdalin in sensitive as well as in the resistant tumor cells. pCDK2, pAkt, and p21 were not detectable in PC3 cells.

Based on the DU145 cell line, pCDK1 was diminished as well, with the strongest effects exerted on the resistant sublines. pCDK2 was reduced in the sensitive and cabazitaxel-resistant cells, whereas the counterpart, Cyclin A, was suppressed in the docetaxel-resistant cells only. In contrast to PC3 cells, Cyclin B was lowered in both the sensitive and drug-resistant DU145 cells. pRictor and pRaptor were also downregulated in the presence of amygdalin (all cell sublines), whereas diminished pAkt was seen in sensitive and docetaxel-resistant but not in the cabazitaxel-resistant cells. P27 was elevated in all cell sublines.

Considerable alterations following amygdalin exposure were detected in LNCaP cells (both sensitive and drug-resistant sublines). CDK1 and 2 (both total and phosphorylated) along with the respective partners, Cyclin A and B, were considerably decreased. Phosphorylated Rictor, Raptor, and Akt were additionally suppressed. Instead, p21 was increased in the resistant cells. P27 was elevated both in the sensitive and docetaxel-resistant LNCaP cells but not in the cabazitaxel-resistant ones, where this protein was diminished.

### 3.4. Modulation of Adhesion and Invasion by Amygdalin

**Figure 6:**
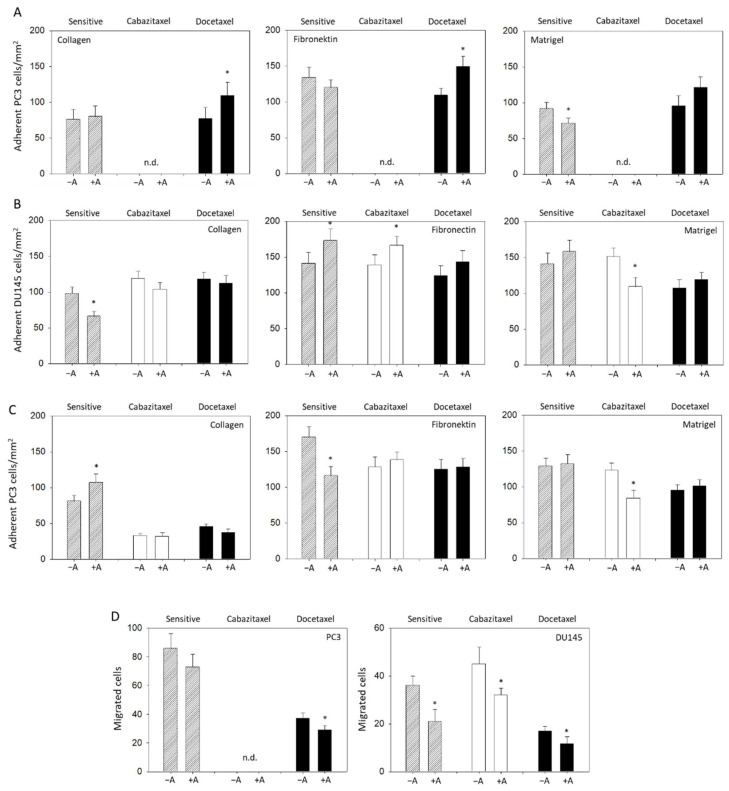
Adhesion of drug-sensitive and drug-resistant PC3 (**A**), DU145 (**B**), and LNCaP cells (**C**) to immobilized collagen, fibronectin, or matrigel. Tumor cells were either treated with amygdalin for 24 h (+A) or remained untreated (−A). (**D**) Effect of amygdalin (+A) on chemotactic migration of PC3 and DU145 cells. Controls were without amygdalin (−A). Bars indicate standard deviation (SD). * indicates significant difference to the corresponding control. n = 5. n.d.: not done.

A moderate influence of amygdalin on tumor cell adhesion behavior was detected. Parental PC3 cells attached at a minor rate to matrigel when treated with amygdalin, compared to the control, whereas the binding to collagen and fibronectin was not altered. Adhesion of docetaxel-resistant PC3 cells to matrigel was not influenced; however, adhesion to collagen and fibronectin was enhanced by amygdalin (Figure 6A). The adhesion behavior of DU145 cells to the matrix proteins was different to the behavior of PC3 cells. Binding of the sensitive cells to collagen was diminished, but binding to fibronectin was enhanced. Furthermore, adhesion of cabazitaxel-resistant DU145 to fibronectin was augmented, whereas binding to matrigel was suppressed by amygdalin exposure. Binding of docetaxel-resistant DU145 cells was not altered by amygdalin (Figure 6B). Contrary to the sensitive DU145 cells, binding of sensitive LNCaP to collagen was elevated, but binding to fibronectin was reduced. The contact of drug-resistant LNCaP with either collagen or fibronectin was not altered, while that of cabazitaxel-resistant LNCaP to matrigel decreased (treated versus nontreated cells) (Figure 6C).

Invasion assays were performed on DU145 and PC3 but not on LNCaP cells that did not demonstrate any chemotactic activity. In this matter, chemotactic movement of PC3 (docetaxel-resistant) and of DU145 (sensitive, cabazitaxel-resistant, and docetaxel-resistant) was significantly downregulated by amygdalin (Figure 6D).

### 3.5. Amygdalin Acts on Cytoskeletal Proteins and Integrin β1

**Figure 7:**
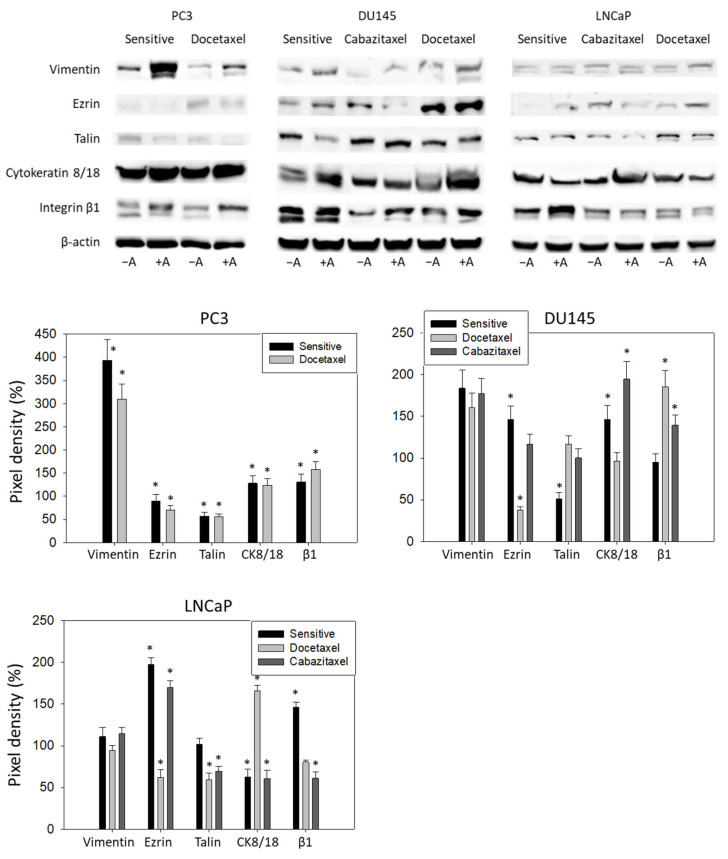
Up: Western blot of cytoskeletal proteins and integrin β1 from drug-sensitive and drug-resistant PC3, DU145, and LNCaP cells. Tumor cells received either amygdalin (+A) or cell culture medium alone (−A) for 24 h. β-actin served as the internal control. One representative from three separate experiments. Down: The ratio of protein intensity/β-actin intensity expressed as a percentage of the controls, set to 100%. Error bars indicate standard deviation, n = 3. * indicates significant difference to controls. Original blots see supplementary file S1.

Amygdalin distinctly elevated the vimentin protein level in PC3 (both sensitive and docetaxel-resistant) and DU145 cells (sensitive, cabazitaxel-resistant, and docetaxel-resistant). The same effect was seen in docetaxel-resistant LNCaP cells (Figure 7). Ezrin was diminished in the sensitive and docetaxel-resistant PC3, and in cabazitaxel-resistant DU145 and LNCaP cells. However, Ezrin was elevated in sensitive DU145 and LNCaP as well as in the docetaxel-resistant LNCaP cells upon amygdalin treatment. A decrease in talin was observed in the sensitive and docetaxel-resistant PC3, in sensitive DU145, and in cabazitaxel- and docetaxel-resistant LNCaP cells. The intermediate filament cytokeratin 8/18 was found to be upregulated by amygdalin in PC3 (sensitive and docetaxel-resistant), sensitive and docetaxel-resistant DU145, and cabazitaxel-resistant LNCaP cells. However, it was downregulated in sensitive and docetaxel-resistant LNCaP cells. Finally, integrin β1 was enhanced in PC3 (sensitive and docetaxel-resistant), cabazitaxel- and docetaxel-resistant DU145, and sensitive LNCaP cells.

## 4. Discussion

Optimal drug sequencing for males with mPCa beyond progression on or after cytotoxic treatment with taxanes remains debatable. In a docetaxel-pretreated mCRPC, a recent meta-analysis of randomized clinical trials utilizing abiraterone acetate, enzalutamide, cabazitaxel, or radium-223 yielded a comparable efficacy for prolongation of overall survival (OS), yet benefits for enzalutamide for extension of time to PSA progression [15]. At the same time, a drug class switch to second-generation hormone therapy compared favorably to other therapies in terms of progression-free survival on subsequent treatment (PFS2) in men with mHSPC, who progressed after upfront docetaxel combined with ADT in a contemporary multicenter real-world data assessment [16]. Since OS has been shown to lie in the range of only 10–15 months in a postdocetaxel mCRPC community setting even with either cabazitaxel- or androgen receptor targeting agents as the second-line treatment [17], an unmet medical need exists for the identification of novel compounds lacking a noteworthy cross-resistance to taxanes, hence qualifying either as their combinable partner, or as a subsequent approach, or both. We present evidence that the natural compound amygdalin exerts substantial anticancer activity in taxane-resistant PCa.

In mammalian cells, amygdalin is metabolized to cytotoxic cyanide by the hydrolytic enzyme β-glucosidase [11]. In turn, the mitochondrial enzyme rhodanese detoxifies cyanide by conversion to thiocyanate. Cyanide’s principle target is cytochrome c oxidase—an indispensable enzyme for operative mitochondrial respiration [18]. Consequently, inhibition of mitochondrial electron transport and aerobic ATP generation would critically impair the function of all cells, especially those with a high bioenergetic turnover, such as cancer cells [18]. In the past, amygdalin has been shown to be a potent inhibitor of cell growth in several neoplasms, e.g., bladder, breast, and pancreatic cancer as well as renal cell carcinoma [19,20,21]. Of note, our group has previously demonstrated a substantial inhibition of cell viability in PCa cell lines upon incubation with amygdalin for 24 h or 2 weeks [14]. The current work confirms a comparable decimation of cell growth by the agent in both parental and taxane-resistant cell lines suggesting the absence of cross-resistance between the compounds at this level. This finding is further corroborated by an impressive reduction of the number of clones and distinct suppression of proliferation in therapy-naïve as well as therapy-resistant cell lines.

Apparently, these effects are substantially -mediated by the G0/G1 phase arrest in response to amygdalin exposure. Interestingly, Park et al. reported on a particularly diminished expression of cell cycle-related genes in human colon cancer cells upon 24 h treatment with amygdalin in their cDNA microarray-based analysis [22]. One of these genes was the FK506 binding protein 12-rapamycin-associated protein 1 (FRAP1/mTOR) possessing serine/threonine kinase activity and promoting G1 phase progression through signaling to p70/S6 kinase and 4E-BP1 [23]. In fact, the activity of Akt and both mTORC1 and mTORC2 complexes including their subunits Raptor or Rictor is required for the G1/S transition [24]. In the current analysis, we noticed a distinct downregulation of phosphorylated Akt, Raptor, and Rictor as well as CDK2 in most scenarios, while the major CDK inhibitor in the G1 phase, p27, was enhanced, explaining mitigation of the G1/S progress. Downregulation of the cell cycle is a known mechanism of cell response to hypoxia [25] and is obvious in our study since amygdalin-derived cyanide restricts aerobe production of ATP to cover energy consumption. Elevated cell proliferation during hypoxia would increase the O_2_ usage fostering hypoxic environment [26]. Previous research by Krtolica et al. on the effect of hypoxia on the cell cycle demonstrated the induction of a reversible cell cycle blockade upon prolonged hypoxia [27]. Flow cytometric analysis revealed an increase in the percentage of cells in the G1 phase and a reduction of the cell number in the S phase following 24 h of hypoxia. In concert with our data, this finding was accompanied by an increased abundance of p27 and a lower activity of CDK2. In our assessment, phosphorylated CDK1 as well as its complex partners Cyclin A and B, which are pertinent for the G2 and M phase entry, was often suppressed highlighting the cell cycle blocking activity of amygdalin at several checkpoints.

Of note, the reduction of phosphorylated Akt upon amygdalin exposure detected in docetaxel-resistant DU145 and LNCaP cells is particularly interesting in the context of PCa. In an immunohistochemical study, Malik et al. demonstrated that the staining intensity for phosphorylated Akt was significantly greater in Gleason grades 8–10 compared to lower grades of PCa [28]. In general, Akt signaling is upregulated in more aggressive PCa subtypes due to the often-encountered PTEN deficiency [29]. In addition, robust evidence exists for the crosstalk between the PI3K-Akt–mTOR pathway and androgen receptor signaling and, particularly, the reciprocal feedback regulation between Akt and androgen receptor [30,31]. In their in vitro study, Kosaka et al. demonstrated that upregulation of phosphorylated Akt during long-term androgen ablation is associated with docetaxel resistance [32]. A combined treatment of docetaxel-resistant cells with a phosphatidylinositol 3-kinase/Akt inhibitor and docetaxel markedly augmented their sensitivity to the latter drug. Other studies revealed that several drugs targeting Akt/PI3K are capable of enhancing the efficacy of docetaxel [29]. As for cabazitaxel, the underlying molecular mechanisms of its resistance have not yet been profoundly elaborated. Nonetheless, Hongo et al. showed an enhanced PI3K/Akt signaling in cabazitaxel-resistant PC3 cells as compared to sensitive ones, while a PI3K/mTOR inhibitor exerted a pronounced antitumor activity in these cells [33]. These data reinforce the promising potential of amygdalin to be applied either concomitantly with or beyond progression on or after taxanes.

While the impact of amygdalin on the binding capacity of PCa cells was incoherent in our work, chemotactic movement was distinctly attenuated by the compound in docetaxel-resistant PC3 as well as in all DU145 cell lines. These findings are in line with the results previously reported for breast, non-small cell lung, bladder, and treatment-naïve prostate cancer cells [34,35,36]. Not only does amygdalin repress cell growth and proliferation, but is it additionally effective in lessening the disseminative capacity of tumor cells. This activity of the compound appears similar in parental and taxane-resistant cells pleading against cross-resistance between amygdalin and docetaxel or cabazitaxel at this level as well.

Concerning the cytoskeleton-related proteins, talin content was mitigated by amygdalin incubation in the majority of cell lines. An elevated expression of talin was associated with unfavorable pathological features and predicted lymphatic metastasis and biochemical recurrence of PCa in an immunohistochemical study by Xu et al. [37]. Notably, the knockdown of talin in DU145 and PC3 cells led to a pronounced suppression of cell migration in the study by Zhang et al. [38]. Remarkably, these data might corroborate our observation of attenuated migration and decreased expression of talin under amygdalin exposure. Previously, Jin et al. reported that talin1 phosphorylation activates β1 integrins that are involved in PCa metastasis to lymph nodes and bones [39]. While the impact of amygdalin on the expression of ezrin and cytokeratin 8/18 was heterogeneous in different cell lines, treatment with this compound was associated with an overexpression of β1 integrin paralleled by that of vimentin in the majority of cell lines in our work.

Although vimentin overexpression has been correlated with augmented tumor growth, invasion, and unfavorable prognosis, the overall relevance of this molecule is not yet clear [40]. With this in mind, Nastały et al. did not find any correlation between vimentin expression and prostate cancer dissemination to the bones [41]. Notably, Hirokawa et al. even noted downregulation of vimentin to be the relevant trigger factor to increase prostate cancer cell migration [42]. Similar ambiguity exists with respect to β1 integrin, which is suggested to be overexpressed or diminished in solid tumors [43]. In their meta-analysis, Sun et al. reported that its enhanced expression predicts a shorter overall survival in lung and breast cancer as well as decreased disease-specific survival in breast and pancreatic cancer [43]. At the same time, the augmented expression of β1 integrin was not related to overall survival in colorectal cancer or melanoma. Surprisingly, some researches argued that reduced β1 integrin protein expression might even be linked to more aggressive breast cancer types [44,45]. Strikingly, recent data even point to integrin β1 as a tumor suppressor in prostate cancer [46]. Obviously, the role of integrin β1 and vimentin is still obscure and requires further evaluation. Upregulation of both proteins may also be interpreted in the context of resistance development counteracting amygdalin activity. In fact, exposing amygdalin to a panel of bladder cancer cell lines suppressed chemotaxis of two cell lines, whereas the crawling activity of one cell line increased [36]. Additional research is warranted to clarify the functional relevance of amygdalin including a broader panel of tumor cell lines. Particularly, the influence of amygdalin on epithelial–mesenchymal transition requires further analysis. Nevertheless, evidence is presented here that amygdalin may block growth and invasion properties of docetaxel- or cabazitaxel-resistant PCa cell lines. Transfer to in vivo studies is now necessary to verify our in vitro results.

## 5. Conclusions

Amygdalin exerts distinct anticancer activity impeding both cell growth and disseminative capacity of taxane-resistant PCa cells. Further scientific efforts are anticipated in order to define the clinical relevance of this compound as an integrative component in the treatment of metastasized PCa.

## Data Availability

All data generated or analyzed during this study are included in this published article.

## Supplementary Materials

The following supporting information can be downloaded at: https://www.mdpi.com/article/10.3390/cancers14133111/s1, File S1: Original Blots.
