# Supplementary material for: Amygdalin Exerts Antitumor Activity in Taxane-Resistant Prostate Cancer Cells
Source: Cancers (Basel). 2022 Jun 24;14(13):3111. doi: 10.3390/cancers14133111 (PMC9265127; doi:10.3390/cancers14133111)
Supplement: Supplementary file 1 [file cancers-14-03111-s001.zip › cancers-1769672-supplementary.pptx]

## Slide 1
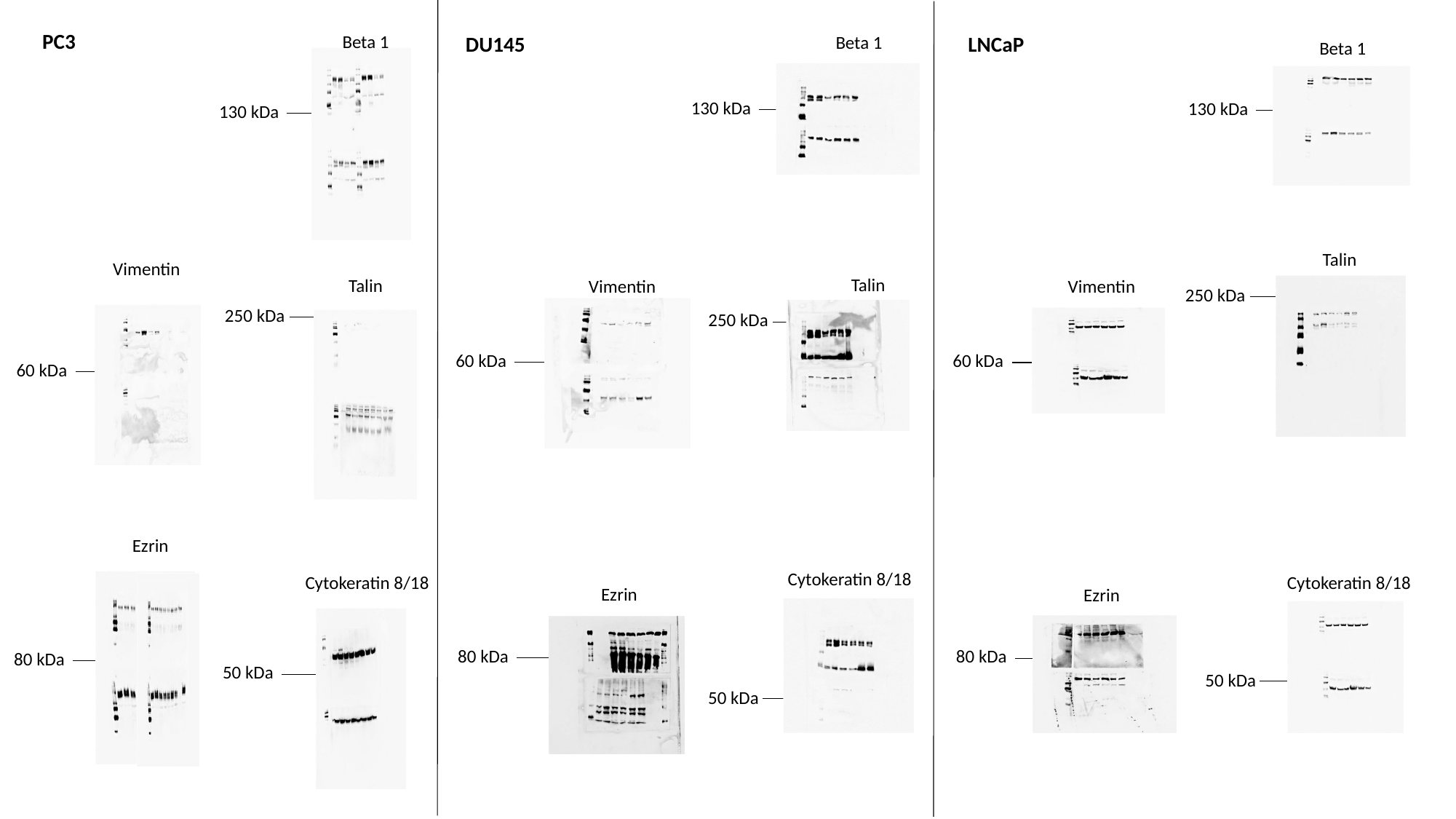

PC3
Beta 1
DU145
Beta 1
LNCaP
Beta 1
130 kDa
130 kDa
130 kDa
Talin
Vimentin
Talin
Talin
Vimentin
Vimentin
250 kDa
250 kDa
250 kDa
60 kDa
60 kDa
60 kDa
Ezrin
Cytokeratin 8/18
Cytokeratin 8/18
Cytokeratin 8/18
Ezrin
Ezrin
80 kDa
80 kDa
80 kDa
50 kDa
50 kDa
50 kDa

## Slide 2
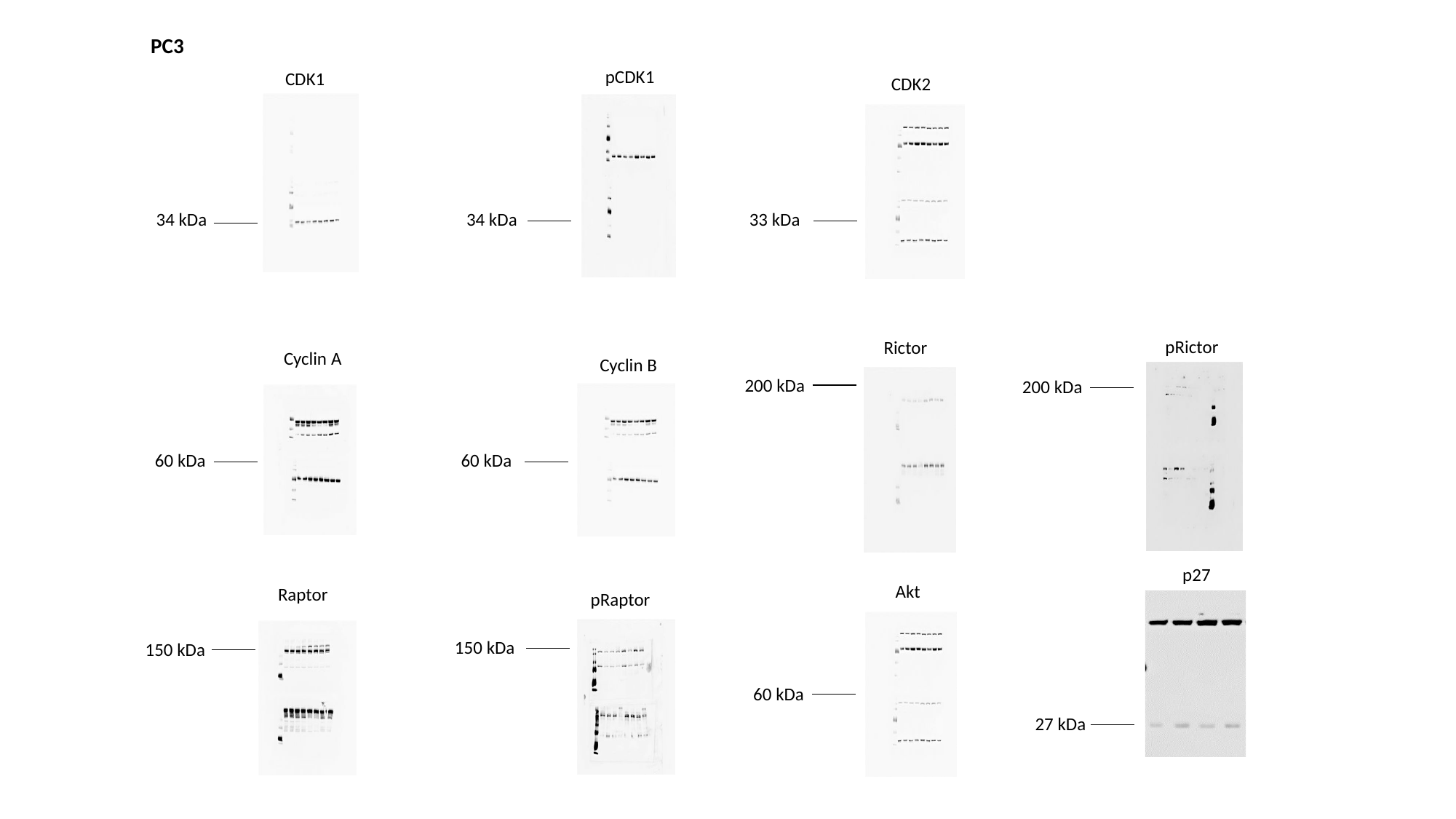

PC3
pCDK1
CDK1
CDK2
34 kDa
34 kDa
33 kDa
pRictor
Rictor
Cyclin A
Cyclin B
200 kDa
200 kDa
60 kDa
60 kDa
p27
Akt
Raptor
pRaptor
150 kDa
150 kDa
60 kDa
27 kDa

## Slide 3
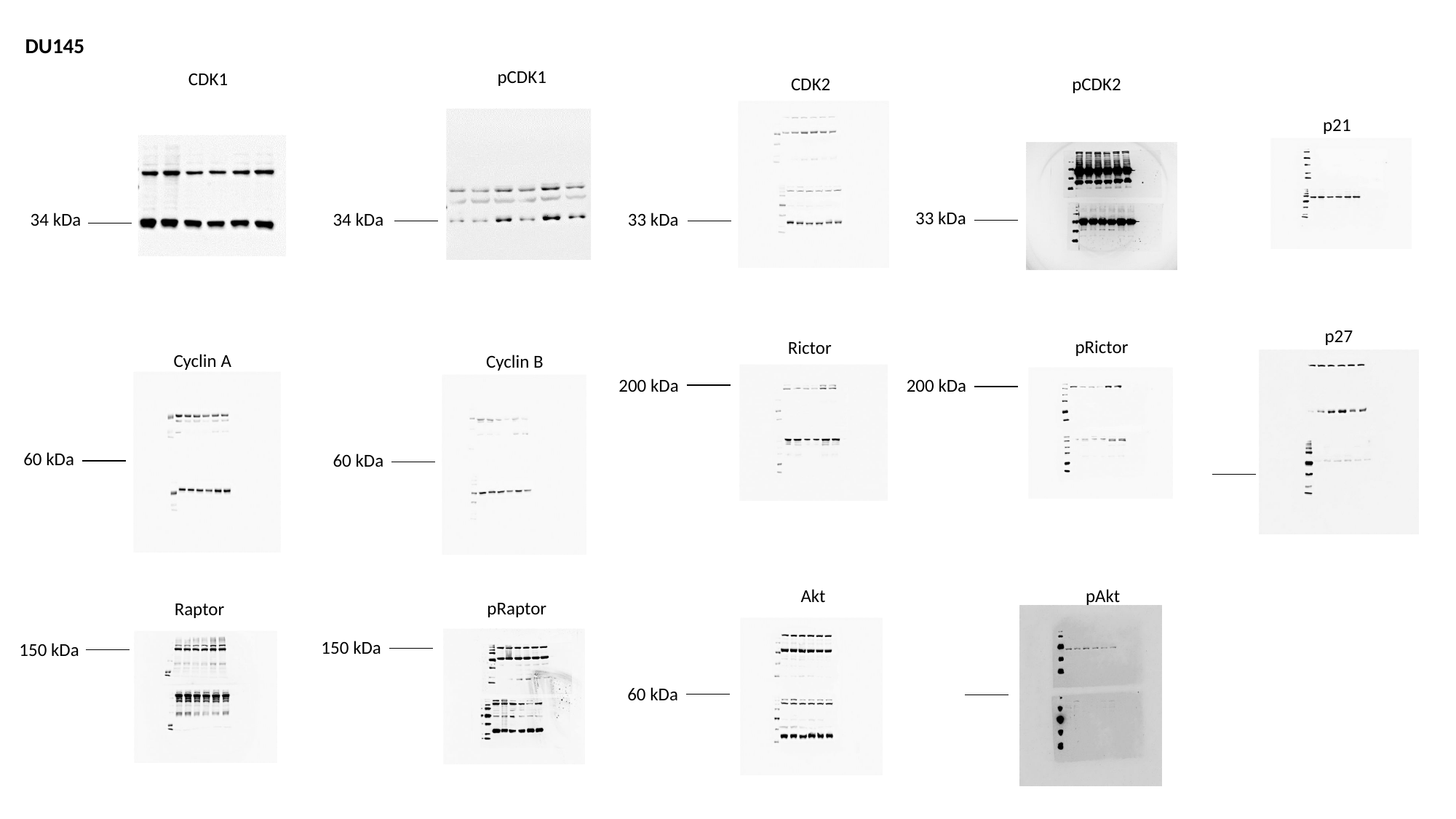

DU145
pCDK1
CDK1
CDK2
pCDK2
p21
33 kDa
34 kDa
34 kDa
33 kDa
p27
pRictor
Rictor
Cyclin A
Cyclin B
200 kDa
200 kDa
60 kDa
60 kDa
Akt
pAkt
pRaptor
Raptor
150 kDa
150 kDa
60 kDa

## Slide 4
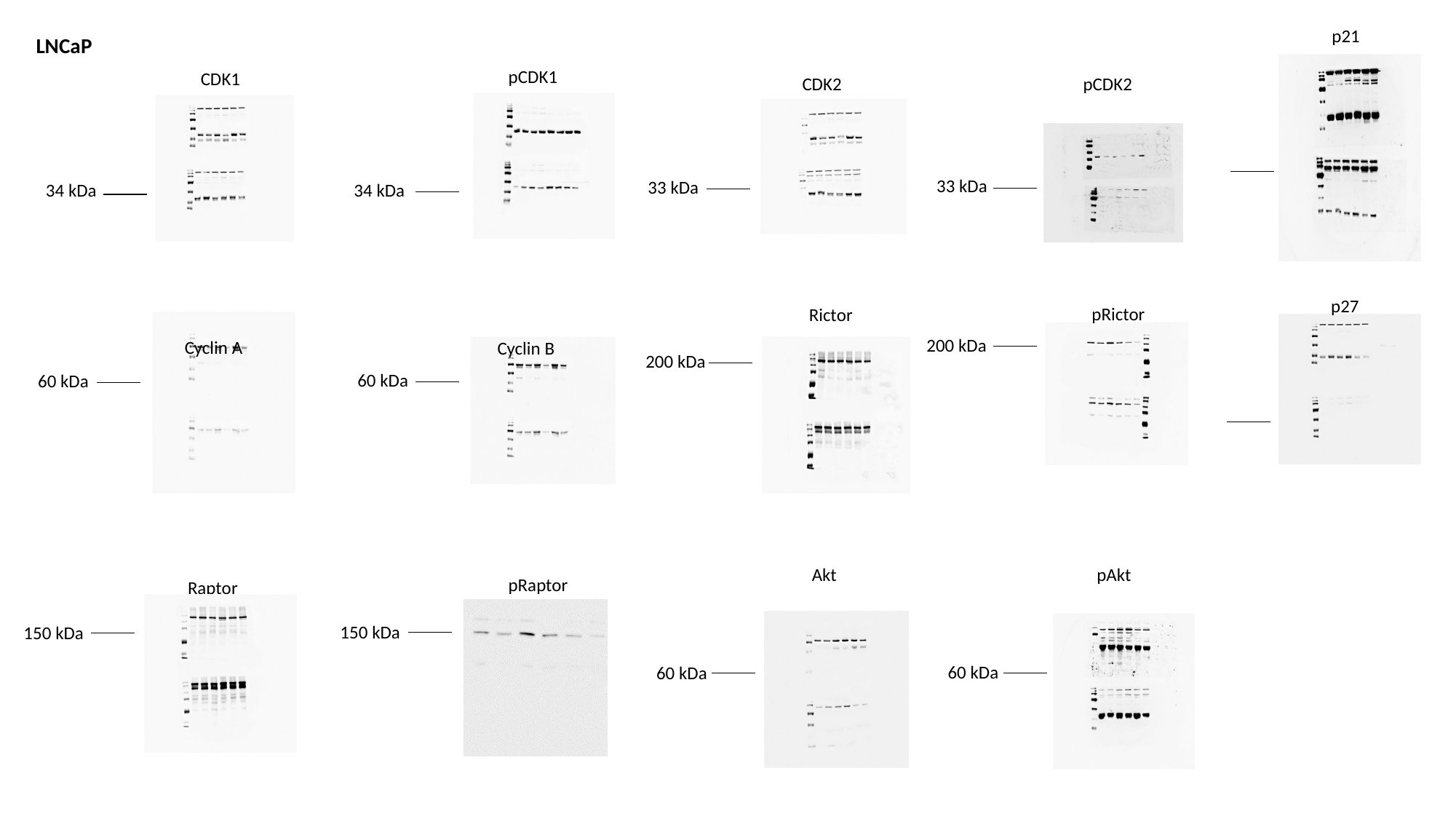

p21
LNCaP
pCDK1
CDK1
CDK2
pCDK2
33 kDa
33 kDa
34 kDa
34 kDa
p27
pRictor
Rictor
200 kDa
Cyclin A
Cyclin B
200 kDa
60 kDa
60 kDa
Akt
pAkt
pRaptor
Raptor
150 kDa
150 kDa
60 kDa
60 kDa
